# Identifying Priority Habitats for Dung Beetle Conservation: Taxonomic and Functional Responses Across a Land‐Use Disturbance Gradient

**DOI:** 10.1002/ece3.72226

**Published:** 2025-09-28

**Authors:** Suk Young Hong, Minwoo Oh, Yoonjeong Heo, Eun Ju Lee

**Affiliations:** ^1^ Seoul National University Gwanak‐gu Seoul Republic of Korea; ^2^ National Institute of Ecology Seocheon‐gun Chungcheongnam‐do Republic of Korea

**Keywords:** conservation biology, functional diversity, functional traits, insect ecology, land‐use change, taxonomic diversity, trophic preference

## Abstract

Anthropogenic activities are causing a decline in dung beetles due to land‐use change and overexploitation. This underscores the need for their conservation, for which identification of high‐priority habitats and associated species is necessary. To achieve this, this study evaluated taxonomic and functional diversity across various land‐use types and dung types. Field experiments were conducted using dung‐baited pitfall traps set up in different land‐use types along a disturbance gradient: built environments, agricultural fields, coniferous forests, and deciduous forests. The traps were baited with wild boar, cattle, and leopard cat dung. With this data, the changes in taxonomic and functional diversity indices were explored. The results indicated that deciduous forests were the most important habitat in most instances, except for small dung beetles and dwellers, which favored agricultural fields. Regarding dung type, wild boar dung was preferred in all cases, with a significantly lower preference for cattle and leopard cat dung. This study utilized multiple indices to identify high‐priority habitats and associated species, emphasizing the significance of deciduous forests, agricultural fields, and wild boar for the conservation of dung beetles.

## Graphical abstract

Anthropogenic land‐use change and resource exploitation are driving declines in dung beetle communities, highlighting the need to identify priority habitats and species for conservation. This study assessed taxonomic and functional diversity of dung beetles across a land‐use disturbance gradient and different dung types using dung‐baited pitfall traps. Results showed that deciduous forests and wild boar dung supported the highest diversity, while small‐bodied species and dwellers preferred agricultural fields, suggesting the importance of deciduous forests, agricultural fields, and wild boar for dung beetle conservation.

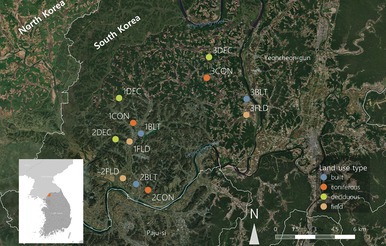

## 1 Introduction

Human activity poses a significant threat to biodiversity. Nearly 20% of vertebrates are classified as “Threatened” (Hoffmann et al. 2010), with their populations having declined by 73% since 1970 (WWF 2024). Although the numbers for invertebrates are more difficult to assess due to the lack of attention, it is predicted that they are experiencing similar losses (Collen et al. 2012). Some of the main causes of this decline include climate change (Sala et al. 2000), pollution (Geiger et al. 2010; Sánchez‐Bayo and Wyckhuys 2019), habitat loss and fragmentation (Newbold et al. 2015; Sala et al. 2000), and overexploitation of fisheries and animals (Dirzo et al. 2014; Niraj et al. 2019).

South Korea is also experiencing biodiversity loss due to rapid economic growth that has caused significant changes to the environment. Urbanization and infrastructure development led to a decrease in wetlands and agricultural environments (National Geography Information Institute 2020), causing habitat loss. Moreover, the modernization of agriculture brought about the introduction of pesticides for crops (Cha et al. 2014) and anti‐parasitic drugs and antibiotics for livestock (William 2012). Pesticides affect non‐target species in higher trophic levels through bioaccumulation (Geiger et al. 2010; Sánchez‐Bayo and Wyckhuys 2019), and anti‐parasitic drugs and antibiotics can affect dung beetles and other coprophagous insects (Bang et al. 2007; Dadour and Cook 1996; Ishikawa and Iwasa 2020), both of which can have significant consequences for biodiversity.

Another significant change was with the forests, which doubled in coverage between the 1950s and 2000s due to natural regeneration and afforestation initiatives (FAO 2020; Park and Lee 2014). The planted areas were mainly monocultures of conifers, which take up 80% of afforested areas and 12.4% of all forests (National Geography Information Institute 2020). One of the primary ecological consequences of monoculture plantations is the loss of biodiversity (Barlow et al. 2007; Bremer and Farley 2010), which is exacerbated when natural forests are replaced. Despite these concerns, the Korea Forest Service (KFS) plans to transform 25,000 ha of forests annually to plant monocultures of fast‐growing trees with high economic value to increase carbon absorption and generate revenue from forest products (KFS 2018, 2021).

Another major change that occurred was among the mammal species. The decline of large carnivores, owing to the destruction of natural habitats and hunting (Jo et al. 2018), caused an increase in water deer ( *Hydropotes inermis* Swinhoe) and wild boar ( *Sus scrofa* Linnaeus) populations (Jo and Baccus 2015). This led to escalated human–wildlife conflicts, prompting a policy that designated them as “harmful wild animals” and provided compensation to poachers for hunting them (ACRC 2020). Hunting of wild boar intensified when African Swine Fever swept through South Korea (Song 2019), which effectively reduced their populations (Lee and Hong 2024). This may have ecological consequences for abiotic (Cha et al. 2012; Pitta‐Osses et al. 2022) and biotic (Cabon et al. 2022; Dovrat et al. 2012) factors due to the cascading effect.

One of the taxa greatly affected by these environmental changes is the dung beetle. Dung beetles are a group of insects belonging to the groups Geotrupidae, Scarabaeinae, and Aphodiinae that rely on vertebrate feces for nutrition and reproduction (Halffter and Edmonds 1982; Hanski and Cambefort 1991). They are sensitive to abiotic factors such as climate and soil properties, as well as biotic conditions such as mammal distributions (Hanski and Cambefort 1991), rendering them vulnerable to habitat change and alterations in mammal community structures (Fuzessy et al. 2021).

Conservation of dung beetles is important not only for dung beetles per se but also for the ecosystem. Dung beetles provide a wide range of ecosystem services, including nutrient cycling, secondary seed dispersal, bioturbation, and pest control (Nichols et al. 2008), highlighting the need for conservation action. Furthermore, they can inform conservation strategies by serving as ecological indicators (Gardner 2010; Nichols and Gardner 2011). However, invertebrate conservation is largely underrecognized (Eisenhauer et al. 2019), emphasizing the need to identify dung beetle species at risk and work towards their conservation.

For the conservation of a group of species, it is important to identify high‐priority habitats and associated species (Gann et al. 2019). To achieve this, assessing taxonomic and functional diversity across different land‐use regimes is crucial. Taxonomic diversity provides information about how diverse a community is based on species identity, while functional traits account for ecosystem functions (Cadotte et al. 2011). Traits often utilized for dung beetles include body length or weight, nesting behavior, daily activity, and trophic preference, offering insights into the ecosystem services provided and their effectiveness (Giménez Gómez et al. 2022).

Dung beetle taxonomic and functional diversity is generally higher in areas with low levels of anthropogenic disturbance (Filgueiras et al. 2022; Frank, Hülsmann, et al. 2017; Nichols et al. 2007; Sullivan et al. 2018). Low disturbance areas, such as primary and secondary forests, display high species richness, evenness, abundance, and functional diversity, along with similar species compositions (Filgueiras et al. 2022; Nichols et al. 2007; Sullivan et al. 2018). However, habitats with higher disturbance, such as pastures and clear‐cuts, exhibit low evenness due to the high abundance of a few species (Nichols et al. 2007). This suggests that areas with low anthropogenic disturbance are high‐priority areas.

However, dung beetles of different functional trait groups were affected differently by changes in habitats. In terms of body size, larger species were more likely to inhabit undisturbed habitats (Barragán et al. 2011; Filgueiras et al. 2022; Larsen et al. 2008), while smaller dung beetles were dominant in disturbed habitats (Filgueiras et al. 2022; Nichols et al. 2007). Regarding nesting method, tunnelers were more abundant in undisturbed habitats, while rollers and dwellers preferred disturbed habitats (Filgueiras et al. 2022). Because large dung beetles are sensitive to environmental change (Barragán et al. 2011; Filgueiras et al. 2022; Larsen et al. 2008) and have the highest dung removal rates (Slade et al. 2007), it can be suggested that habitats supporting large species are important. Additionally, since different functional guilds may contribute differently to ecosystem services (Milotić et al. 2019; Slade et al. 2007), areas with high functional diversity should also be considered.

Another important factor to consider in dung beetle conservation is the biotic interaction with mammals (Torabian et al. 2024), which also significantly influences dung beetle taxonomic and functional diversity (Culot et al. 2013; Nichols et al. 2009). Even the loss of a single mammal species can reduce the richness and abundance of dung beetles (Enari et al. 2013; Estrada et al. 1999). Although the dung of mammal species or feeding guilds, that is, carnivores, herbivores, and omnivores, favored by dung beetles varies across different regions, omnivore dung (Frank et al. 2018; Hanski and Cambefort 1991; Whipple and Hoback 2012) and herbivore dung (Frank et al. 2018; Hanski and Cambefort 1991) are typically preferred over carnivore dung in most cases. In terms of trait groups, the decline of large mammals has been shown to negatively affect large dung beetles (Culot et al. 2013; Fuzessy et al. 2021). Therefore, identifying the mammal species that are important for dung beetle communities is a crucial step toward dung beetle conservation.

Based on the results of previous studies, it was hypothesized that habitat disturbance was related to dung beetle species assemblages and that preferences for specific types of dung exist. The following predictions from these hypotheses were tested. First, low‐disturbance areas, such as deciduous forests and coniferous forests, will exhibit higher richness, abundance, and functional diversity than high‐disturbance areas, represented by built environments and agricultural fields. Additionally, the species composition will be similar in the two low‐disturbance environments. Second, large dung beetles and tunnelers will occur in low disturbance areas, while small dung beetles and dwellers will be present in high disturbance areas in high numbers. Third, omnivore dung and/or herbivore dung will be more attractive to dung beetles, and the size of the mammals will correspond to the size of the dung beetles.

Environmental changes driven by urbanization, modernization of agriculture, forestry practices, and shifting mammal communities in South Korea are among the key factors that have likely altered dung beetle assemblages. Assessing this change is crucial for identifying high‐priority habitats and associated species to inform effective conservation strategies for dung beetles. To this end, field surveys were conducted at sites with varying land‐use types and different mammal dung to examine the dung beetle community structure under each condition. This research aims to enhance our understanding of how dung beetle assemblages change across land‐use types and dung types by using taxonomic and functional diversity. Through this, this study aims to contribute to the conservation of dung beetles and the associated ecosystem.

## 2 Methods

### 2.1 Study Site

**FIGURE 1:**
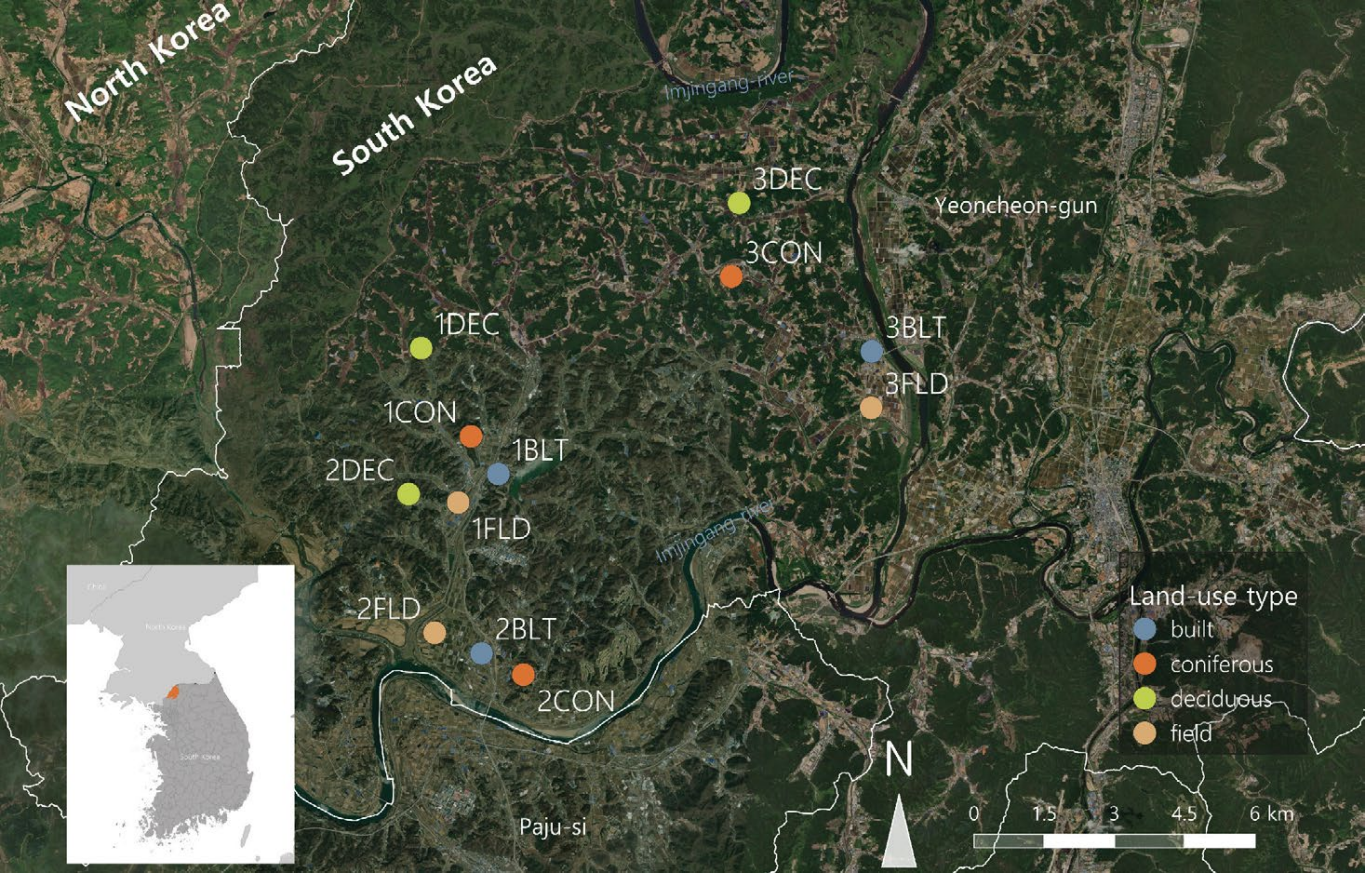
Study area in Yeoncheon‐gun, South Korea. The area borders North Korea to the northwest, and the Imjin‐gang River encircles it on all other sides. Each of the 12 sites is represented by dots, and the color of the dots stands for different land‐use types. Each land‐use type had three sites, with each set of land‐use types grouped into a “region.” The regions are marked with numbers in front of the land‐use types. The Esri World Imagery (Esri 2025) was used as a basemap.

The dung beetles were sampled near the Demilitarized Zone (DMZ) in Yeoncheon‐gun, Gyeonggi‐do Province, South Korea, north of the Imjin‐gang River (Figure 1). This area was part of the Civilian Control Zone (CCZ), where civilian access was limited. Although the restrictions were lifted in 1990, proximity to North Korea inhibited development in the area (Heo and Kim 2008). Currently, this area mainly consists of rice paddies and low hills with secondary oak forests and coniferous forests. Research in the DMZ and CCZ indicates that these areas host a high biodiversity of vertebrates (Cho 2019; Kim and Cho 2005), suggesting a potential for rich dung beetle communities (Nichols et al. 2009). Therefore, this location was chosen due to its relatively low levels of development and potential for high dung beetle diversity.

A total of 12 sites were selected within the study area, comprising three sets of four land‐use types across different levels of disturbance. The land‐use types are built environment, agricultural fields, coniferous forests, and deciduous forests, listed in decreasing order of disturbance. The built environment had traps installed on elementary school grounds, while agricultural fields were located adjacent to rice paddies and croplands. Coniferous forests consisted of *Pinus koraiensis* Siebold & Zucc. (regions one and two) and *Pinus densiflora* Siebold & Zucc. (region three) aged approximately 30–40 years (KFS 2013). Evidence of management could be observed in the preventative measures taken for pine wilt disease and the absence of successional development (personal observation). Deciduous forests were secondary forests with 40–50‐year stands of *Quercus* spp. (KFS 2013). The distance between the sites was at least 900 m, which exceeded the suggested 50–100 m (Mora‐Aguilar et al. 2023) to ensure independence.

### 2.2 Sampling Design and Species Identification

In each site, three dung‐baited pitfall traps were placed, each with a different dung type. They were placed at five‐meter intervals, forming a 10‐m line transect. Sampling was conducted monthly from April to November 2022 and 2023, totaling 16 sampling sessions per site. Dung beetles were identified to species level on‐site. When identification was difficult, photos were taken and/or specimens were collected for identification in the laboratory. Dung beetles were identified using Kim (2012) and Kawai et al. (2005).

There are known limitations to using pitfall traps, as they can oversample species that are highly active and mobile, or particularly responsive to dung bait (Greenslade 1964; Topping and Sunderland 1992), which warrants caution when interpreting results from data collected using pitfall traps. However, pitfall traps were chosen for this study because they are the most widely used method for sampling dung beetles, allowing for comparability and standardization across studies (Mora‐Aguilar et al. 2023). Furthermore, this method is simple, inexpensive, easy to use, and highly adaptable, making it practical for large‐scale or long‐term ecological research (Favila and Halffter 1997; Lobo et al. 1988).

The trap used in this study consisted of a plastic container with a 192 mm diameter and 236 mm depth, a funnel with a top diameter of 200 mm, a bottom diameter of 35 mm, and 125 mm depth, and a plastic dish with a 230 mm diameter. Instead of the usual preservative liquids, the plastic container was filled with approximately 5 mm of soil (Lobo et al. 1988) to avoid killing dung beetles. It was then capped with the funnel to prevent dung beetles from escaping. The trap was buried so that the top of the funnel was level with the ground. The plastic dish served dual purposes of preventing rain from entering the trap and securing the dung bait in place. It was elevated about 50–70 mm from the ground using steel wires. Approximately 105 ± 5 g of pre‐frozen dung was placed inside disposable broth bags and secured under the plastic dish with steel wires.

Each sampling session lasted approximately 72 h. This exceeded the suggested sampling period of 48 h (Mora‐Aguilar et al. 2023), but since frozen dung remained attractive to dung beetles for 3 days (Bezanson et al. 2021), and because dung of varying ages may attract different species (Aruchunnan et al. 2015), 72 h was chosen. This timeframe was expected to capture late‐coming dung beetles while mitigating the negative effects of rainfall, rodents, and birds.

The three mammals were selected based on the following criteria. First, they must be relatively common in the agricultural landscape of South Korea. Second, they need to belong to different trophic guilds, that is, omnivores, herbivores, and carnivores. Third, their dung must be available in large quantities. The three species that met these criteria were wild boar (omnivore), cattle ( *Bos taurus* Linnaeus, herbivore), and leopard cat ( *Prionailurus bengalensis* Kerr, carnivore).

Dung from boar and cattle was collected in early March for 2022 (monthly mean temperature at collection sites: 3.9°C) and late February for 2023 (monthly mean temperature at collection sites: −1.7°C) to be used in the same year. These colder months were chosen to minimize the loss of attractiveness to dung beetles, which typically decline after 2–3 days in the field (Bezanson et al. 2021).

Boar dung was collected at a farm in Taebaek‐si, Gangwon‐do province. The boars were fed apples, and it is highly likely that they also fed on insects. Boar dung was collected over two consecutive days, mixed for homogenization, and portioned into plastic bags on‐site. Although attractiveness may begin to decrease after 1 day, the low temperatures during dung collection, and the small decline in attractiveness by the second day (Bezanson et al. 2021), suggest that a two‐day collection period was acceptable. Cattle dung was collected at Seoul National University Animal Farm in Pyeongchang‐gun, Gangwon‐do province. Dung from dairy cows, which were fed hay and other grain‐based feed, was collected. The freshest pats were selected, mixed to homogenize consistency, and then portioned into plastic bags on‐site.

Leopard cat dung was collected at Seoul Zoo between the years 2022 and 2023. The leopard cats were fed chicken, mice, and other meats. Dung was collected daily by the zookeepers and kept in a freezer for up to several months at the zoo. When enough dung was collected, it was brought to the lab, half‐thawed, and portioned into plastic bags. All dung types were kept in a freezer at −24°C until use, as frozen dung is easy to store, and may be more attractive to dung beetles for the first 2–3 days (Bezanson et al. 2021).

### 2.3 Data Analysis

#### 2.3.1 Sampling Completeness

To assess the adequacy of sampling effort and the reliability of observed species richness, coverage‐based rarefaction and extrapolation curves were generated using the iNEXT package (Hsieh et al. 2024). Land‐use type and dung type were the variables used to evaluate sampling completeness.

#### 2.3.2 Species Composition

Variation in dung beetle composition across each variable was assessed using permutational analysis of variance (PERMANOVA). The variables included land‐use type, dung type, year, and region. To avoid over‐representation of rare species, those with fewer than three observations were excluded. PERMANOVA was performed using the vegan package (Oksanen et al. 2024).

#### 2.3.3 Taxonomic Diversity

Three diversity indices were employed to assess taxonomic diversity: abundance, estimated species richness, and total species richness. Abundance represented the total number of individuals per species. For the estimated species richness (referred to as “richness” hereafter), the Chao1 index was utilized to account for undiscovered rare species, calculated using the iNEXT package (Hsieh et al. 2024). Total species richness represented the count of all species across each of the land‐use types and dung types. It was visualized using Venn diagrams created with the ggVennDiagram package (Gao and Dusa 2024).

#### 2.3.4 Functional Traits and Diversity

Two functional traits were explored: size and nesting method. Size was categorized into small (≤ 5.5 mm), medium (> 5.5 mm and ≤ 10 mm), and large (> 10 mm) depending on dung beetle body length, which was gathered from Kim (2012). The mean value of the minimum and maximum body length was calculated for categorization. The 10 mm cutoff was chosen to align with previous studies that have employed a binary size classification of small and large based on a 10 mm boundary (Barragán et al. 2011; Hanski and Cambefort 1991; Navarrete and Halffter 2008). To account for the high number of smaller‐bodied species in South Korea, an additional threshold of 5.5 mm was introduced to divide these species into two approximately equal groups.

This three‐size classification was designed to better reflect the distribution of body sizes in the regional dung beetle fauna and facilitate analysis of potentially nonlinear ecological responses, although the thresholds are inherently arbitrary. It also broadly corresponds to taxonomic groupings: small species consisted mainly of the subfamily Aphodinae and genus *Caccobius*, while many medium species belonged to the genus *Onthophagus*, and large species included the family Geotrupidae and subfamily Scarabaeinae (Table A1).

For the nesting method, species were assigned one of three categories: dweller, roller, and tunneler, depending on how dung beetles utilize their dung resources. Dwellers lay their eggs inside the dung pat, while tunnelers create tunnels beneath the dung pat where they store dung and lay their eggs. Rollers, on the other hand, pack dung into balls to roll away from the dung pat to bury and lay their eggs.

For functional diversity, three indices were examined. Functional richness (FRic) measures the volume of trait space occupied by the species in a community, reflecting the range of functional traits. Functional dispersion (FDis) represents the abundance‐weighted mean distance of species to the community centroid in trait space, capturing trait variability. Quadratic entropy (RaoQ) quantifies the abundance‐weighted sum of pairwise functional dissimilarities among species, integrating both trait differences and species abundances. These indices were calculated using size and nesting strategy as functional traits, with the FD package (Laliberté et al. 2014).

#### 2.3.5 Generalized Linear Mixed Models (GLMMs)

For all species indices, the variation in dung beetle community structure was examined using GLMMs with the glmmTMB package (Brooks et al. 2017). The variables land‐use type and dung type served as fixed variables, and the abundance, richness, and functional diversity values were used as the response variables. The months and site ID were used as random variables to account for the seasonal variability and to control for potential pseudo‐replication due to repeated measures within sites. Additional variables, such as year and region, were included as fixed or random variables depending on the Akaike Information Criterion (AIC) values and residual diagnostics.

Different distributions were selected for the diversity indices based on the characteristics of the response variable. For richness and FRic, which consisted of positive, non‐integer values, a Gamma distribution with the log link function was used. Abundance, being count data with overdispersion, was modeled using a negative binomial distribution. For functional trait richness, a Tweedie distribution with the log link function was employed because it had positive continuous values with many zeros. Functional trait abundance was modeled with a negative binomial distribution, as with taxonomic abundance. Finally, FDis and RaoQ, which were continuous and included zeros, were modeled using a Gaussian distribution.

After the models were constructed, residuals were evaluated to assess model fit using the DHARMa package (Hartig 2024). Next, pairwise comparisons among levels were performed using the emmeans package (Lenth 2024). Then, the results were visualized using the interactions package (Long 2024). Only FRic by land‐use type was visualized using the estimated marginal means (emmeans) because they better reflect the raw data distribution.

As a final step, spatial autocorrelation in the site‐level random intercepts was checked using Moran's *I*, with sites within 2500 m defined as neighbors. Moran's *I* values ranged from −0.374 to 0.404 across all diversity metrics and trait groups, and none of the tests were statistically significant (*p* > 0.05). This suggests that the estimated site‐level effects did not exhibit spatial autocorrelation. The sf package was used for handling spatial data, and the spdep package for calculating Moran's *I* (Pebesma and Bivand 2023). All analyses for this study were conducted using R (R Core Team 2023).

## 3 Results

### 3.1 General Results

**FIGURE A1:**
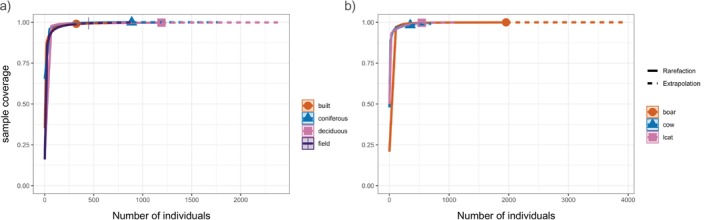
Sample completeness curves for (a) land‐use type and (b) dung type. Sample coverage reached over 98% for all variables at all levels.

In the field experiment, 2847 individuals from 24 species were captured. Of these, 14 species belonged to the subfamily Scarabaeinae, nine species to Aphodiinae, and one species to the family Geotrupidae. Within the Scarabaeinae, the genus *Onthophagus* was most numerous with nine species, and for Aphodiinae, the genus *Aphodius* was most numerous with seven species. In terms of functional groups by size, 10 small, 11 medium, and three large species were identified. By nesting method, there were nine dwellers, one roller, and 14 tunneler species (Table A1). The sampling completeness curves reached an asymptote near 1.0 for all cases examined, suggesting that the sampling effort was adequate for detecting most of the dung beetle species assemblage (Figure A1).

### 3.2 Land‐Use Type

**FIGURE 2:**
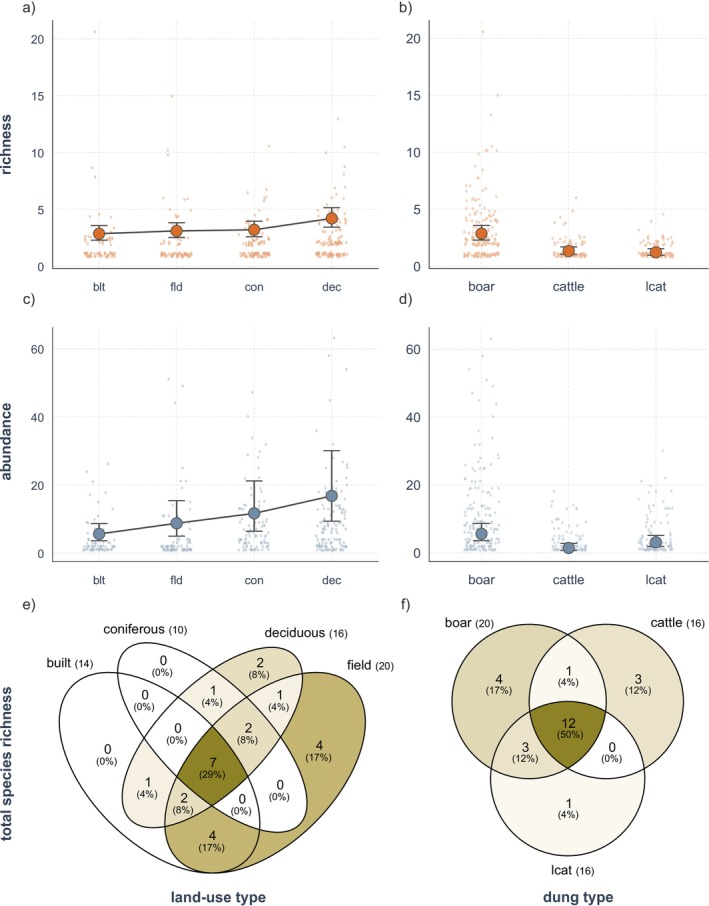
Taxonomic diversity of dung beetles by land‐use type and dung type. The richness across (a) land‐use types, (b) dung types is represented by graphs with orange dots, while abundances across (c) land‐use types and (d) dung types are illustrated with blue dots. Total species richness by (e) land‐use type and (f) dung type show the total number of species found at each level in parentheses, while the number within the circles denote the number of species and the percentage compared to the total species (24). “blt” stands for built environment, “fld” for agricultural field, “con” for coniferous forest, and “dec” for deciduous forest.

**FIGURE 3:**
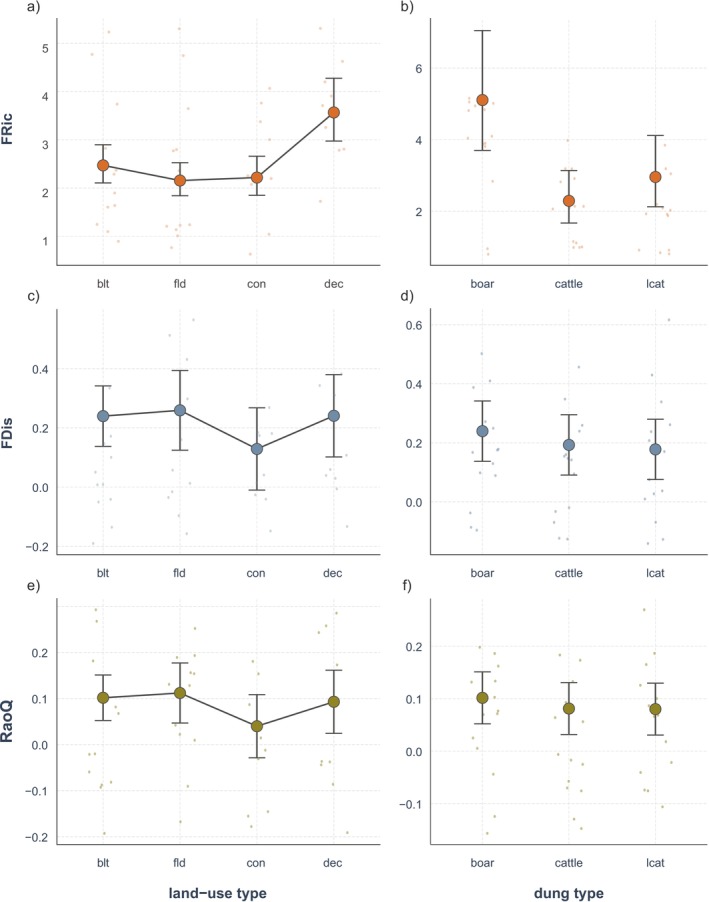
Functional diversity of dung beetles by land‐use type and dung type. The values for functional richness (FRic; a, b), functional dispersion (FDis; c, d), and functional entropy (RaoQ; e, f) are illustrated. “blt” stands for built environment, “fld” for agricultural field, “con” for coniferous forest, and “dec” for deciduous forest.

The species composition changed significantly according to land‐use types, accounting for 25.2% of the variation in dung beetle species (*F* = 8.196, *R* ^2^ = 0.252, *p* = 0.001; Table A2). Taxonomic diversity indicated that deciduous forests had the highest richness and abundance, as well as total species richness (Figure 2, Table A3) and functional richness (Figure 3, Table A4) of dung beetles. Coniferous forests had low species richness, similar to built environments and agricultural fields, but had the second‐highest abundance (Figure 2, Table A3), due to the high number of generalist species *Onthophagus fodiens* (Table A1). Species assemblages in coniferous forests were a subset of those in deciduous forests (Figure 2). Both built environments and agricultural fields displayed low richness and abundance (Figure 2, Table A3). However, total species richness was highest at agricultural fields with 20 species and lowest in coniferous forests with 10 species (Figure 2). Coniferous forests also had the lowest functional dispersion and quadratic entropy, although at a marginally significant level (Figure 3, Table A4).

**FIGURE 4:**
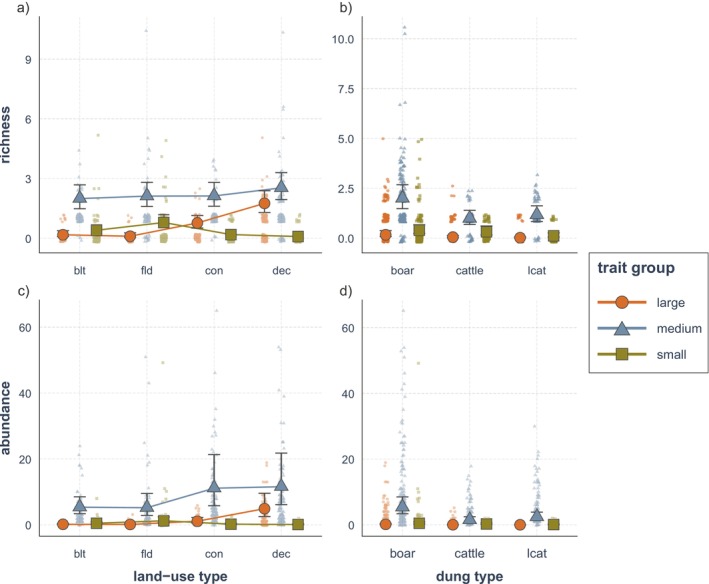
Dung beetle richness and abundance across land‐use types and dung types by size. Dung beetles were categorized into large, medium, and small depending on their body length. Changes across land‐use type (a, b) and dung type (c, d) are shown. The model was fitted with land‐use type, dung type, and trait group as fixed variables, while month and site ID were treated as random variables. The interactions between land‐use type and dung type with trait groups were considered in the model. “blt” stands for built environment, “fld” for agricultural field, “con” for coniferous forest, and “dec” for deciduous forest.

**FIGURE 5:**
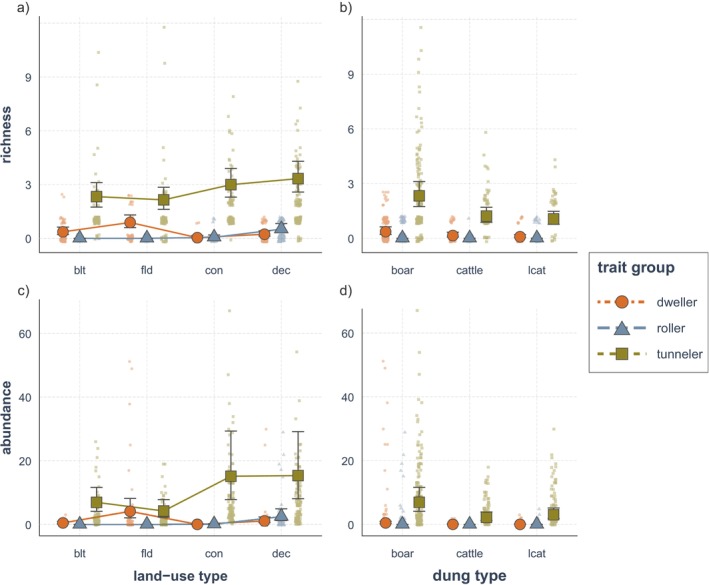
Dung beetle richness and abundance across land‐use types and dung types by nesting method. The dung beetles were categorized into dwellers, rollers, and tunnelers depending on how they utilized dung. Changes across land‐use type (a, b) and dung type (c, d) are shown. The model was fitted with land‐use type, dung type, and trait group as fixed variables, while month and site ID were treated as random variables. The interactions between land‐use type and dung type with trait groups were considered in the model. “blt” stands for built environment, “fld” for agricultural field, “con” for coniferous forest, and “dec” for deciduous forest.

Dung beetles with different functional traits exhibited varying responses to different land‐use types. Deciduous forests supported the highest richness and abundance of large dung beetles (Figure 4, Tables A5 and A6), as well as rollers and tunnelers (Figure 5, Tables A7 and A8). The abundance of medium‐sized dung beetles was greatest in forest habitats, but richness remained similar across all land‐use types (Figure 4). Tunneler dung beetles were also associated with both types of forests (Figure 5). For rollers, the majority (97.4%) of dung beetles were found in deciduous forests (Table A1). In contrast, small dung beetles and dwellers preferred agricultural fields, where the highest richness and abundance of these functional trait groups are observed (Figures 4 and 5, Tables A5, A6, A7, A8).

### 3.3 Dung Type

The species composition changed significantly according to dung types, accounting for 13.0% of the variation in dung beetle species (*F* = 5.546, *R* ^2^ = 0.130, *p* = 0.001; Table A2). Boar dung scored the highest among richness, abundance, functional diversity, and functional traits explored in this study, while cattle dung and leopard cat dung were similarly less preferred in most cases (Figures 2, 3, 4, 5, Tables A3, A4, A5, A6, A7, A8).

Comparisons between leopard cat dung and cattle dung showed that leopard cat dung attracted a higher abundance of dung beetles compared to cattle dung (Figure 2, Table A3), primarily due to the high abundance of *O. fodiens* and *Onthophagus atripennis* Waterhouse found at leopard cat dung (Table A1). Moreover, total species richness was significantly higher at leopard cat dung compared to cattle dung in agricultural fields (Table A1). For functional traits, small dung beetles exhibited high richness and abundance at cattle dung, similar to that for boar dung, whereas medium dung beetles were more abundant at leopard cat dung than at cattle dung (Figure 4, Tables A5 and A6). By nesting type, only rollers showed a higher preference for leopard cat dung over cattle dung (Tables A7 and A8).

## 4 Discussion

### 4.1 Land‐Use Type

As hypothesized in the first prediction, the results demonstrated that species richness, abundance, and functional diversity were highest in sites with the lowest disturbance, that is, deciduous forests. Additionally, sites with low disturbance had similar compositions, as the dung beetle community in coniferous forests was a subset of that within deciduous forests. However, the total species richness and functional diversity of coniferous forests were comparable to or lower than those in high‐disturbance areas. In accordance with the second prediction, large dung beetles and tunnelers favored low‐disturbance areas, while small dung beetles and dwellers preferred higher‐disturbance areas.

The preference for deciduous forests highlights their role as critical habitats for dung beetles. Their relatively undisturbed status can be attributed to several factors, including proximity to North Korea, the designation of the CCZ, and the potential threat of land mines. In addition, their status as secondary forests may contribute to high richness and abundance, as they resemble primary forests, which had high species richness, abundance, and functional diversity, albeit to a lesser extent (Audino et al. 2014; López‐Bedoya et al. 2022; Nichols et al. 2007).

Trait groups particularly associated with deciduous forests include large dung beetles and rollers. Large, forest‐dwelling dung beetles are more sensitive to anthropogenic disturbances (Barragán et al. 2011; Larsen et al. 2008), which may explain their presence in the less disturbed deciduous forests of the study area. Notably, two of the large forest dung beetles, *Copris tripartitus* Waterhouse and *Onthophagus rugulosus* Harold, are listed in the Korean Red List of Endangered Species (Bae et al. 2013). Roller species were found almost exclusively in deciduous forests, unlike in the other parts of the world, where they preferred open environments, such as agricultural areas (Filgueiras et al. 2022; Lobo 2001) and savannas (Kunz and Krell 2011). Although this may be due to the extinction of other roller species in South Korea: *Gymnopleurus mopsus* and *Scarabaeus typhon*, which were recorded in open habitats in the past (Bae et al. 2013).

Compared to deciduous forests, coniferous forests are species‐poor environments with low functional diversity, despite consisting of native trees that are similar in age to those in deciduous forests. Continued management of coniferous forests may have acted as a disturbance, leading to species loss and an increase in the abundance of a few generalists, as seen in the case of the Neotropics (Nichols et al. 2007; Rivera et al. 2023). Additionally, coniferous forests differ in soil texture, acidity, and understory plant species compared to deciduous forests (Hızal et al. 2013; Verstraeten et al. 2013), which can influence dung beetle community structures (Hanski and Cambefort 1991).

Therefore, KFS's plan to increase conifer plantations may pose a potential risk to dung beetle communities by increasing disturbance and altering environmental conditions. Furthermore, although the total area of conifer monocultures constitutes only about 12.4% of all forests in South Korea (National Geography Information Institute 2020), increasing plantations may cause fragmentation of deciduous forests, which can also influence dung beetle communities (Horgan 2007). While additional studies need to be conducted in South Korea to assess the specific effects of plantations, the potential consequences of habitat alteration and fragmentation suggest the importance of considering dung beetle diversity in forest planning and management.

Agricultural fields saw the highest total species richness, high functional diversity compared to coniferous forests, and a high richness and abundance of small and dweller species. One possibility is that intermediate levels of disturbance led to the diversification of dung beetles in this environment, aligning with the intermediate disturbance hypothesis (Connell 1978). Another possibility is that dung beetles in South Korea may have adapted to coexist with humans in these agricultural settings. In Southern Europe, the highest species richness was also observed in open environments, likely due to the long history of pastures in the region (Tocco et al. 2013). Rice paddies and croplands form part of Korea's traditional agricultural landscape, which dates back approximately 3000 years (Ahn 2010) and may have provided suitable habitats that dung beetles have adapted to over time.

The high species richness and abundance of small dung beetles in agricultural fields may be partially explained by their preference for cattle dung, as cattle are commonly raised in these areas. Furthermore, small dung beetles may be better adapted to the open environment microhabitat, where higher daytime temperatures help offset the thermoregulatory limitations associated with low endothermy (Giménez Gómez et al. 2020). Dwellers, which breed and feed within dung pats, may also be well‐suited to open habitats, as the microclimate inside dung pats provides protection from high temperature and low humidity environments (Nervo et al. 2021). The patterns in taxonomic and functional trait diversity suggest that certain dung beetle groups may be particularly well adapted to anthropogenic habitats, potentially contributing to the high diversity observed in agricultural landscapes.

However, dung beetles in these environments are threatened due to changes in agricultural practices. Cattle and other livestock are kept in barns, fed grain‐based diets, and medicated with antiparasitic drugs like ivermectin, which limits resources for dung beetles and affects their reproduction (Bang et al. 2007; Dadour and Cook 1996; Ishikawa and Iwasa 2020). These pressures have led to the regional extinction of *Gymnopleurus mopsus* Pallas, a large (body length = 11.5 mm) roller species that inhabited field environments (Bae et al. 2013), demonstrating that changes to agriculture were a significant disturbance for dung beetles. Additionally, the use of pesticides in agricultural landscapes may further affect dung beetles (Cavallaro et al. 2023; Imelda Martínez et al. 2017), although they are unlikely to be the primary targets.

The built environment had the lowest richness and abundance across most diversity indices. This is likely due to high levels of disturbance and hostile conditions (Ramírez‐Restrepo and Halffter 2016). However, the relatively high richness and abundance of small and dweller species in these areas may result from spillover from adjacent agricultural fields or reflect their general tolerance for, or adaptation to, the disturbed status of built environments. Regardless of the underlying cause, low diversity in built environments warrants greater attention to the ecological impacts of urban sprawl into agricultural landscapes, as it may have important consequences for dung beetle communities.

### 4.2 Dung Type

As indicated in the third prediction, all dung beetles and their trait groups displayed a marked preference for dung from the omnivorous wild boar. However, the prediction that dung beetle size corresponds to the size of the mammal whose dung they prefer was not supported. Contrastingly, small dung beetles showed a strong preference for large mammal (cattle) dung over small mammal (leopard cat) dung, while medium dung beetles preferred small mammal dung over large mammal dung. These findings suggest no clear relationship between dung beetle size and mammal size.

The results support earlier observations that dung beetles are often attracted to omnivore dung, such as humans, monkeys, and boars (Frank et al. 2018; Whipple and Hoback 2012). In Japan, the dung of Japanese macaques ( *Macaca fuscata* ) is a key resource for dung beetles, and their absence has been linked to reduced dung beetle abundance (Enari et al. 2013). Similar patterns have been reported for howler monkeys in Mexico (Estrada et al. 1999). In this context, the strong affinity of dung beetles for boar dung in this study suggests that it is a key resource for dung beetles in South Korean ecosystems. Given this, increasing hunting pressure on wild boars in South Korea could have unintended ecological consequences, especially for dung beetle communities that rely on their dung.

Compared to boar dung, both cattle dung and leopard cat dung were generally less attractive to dung beetles. In other parts of the world, the results with cattle dung were mixed (Enari et al. 2013; Frank et al. 2018). In South Korea, cattle dung was not a preferred resource except for small dung beetles. However, this may have been different in the past, before the modernization of livestock management. Previously, cattle were allowed to graze freely, whereas now they are fed grain‐based feed. Perera et al. (2022) reported that dung from horses fed on pasture was more attractive to dung beetles than dung from horses fed lucerne hay. Similarly, dung from freely grazed cattle may attract a wider variety of dung beetles. The shift to grain‐based feed and the use of antiparasitic drugs likely altered the dung beetle community associated with cattle dung.

Carnivore dung is generally the least preferred by dung beetles (Hanski and Cambefort 1991), although a high abundance of some species may be observed (Whipple and Hoback 2012). This was also the case for leopard cat dung in South Korea, where medium‐sized beetles and tunnelers were observed in greater abundance than at cattle dung, exhibiting functional variation in preference.

In summary, boar dung was the most preferred resource for most dung beetles, while the preference for cattle dung and leopard cat dung varied across different dung beetle groups. However, caution is needed in generalizing these findings to the trophic guild level, as only one representative mammal dung was tested per trophic guild. For example, dung from herbivores such as water deer or other omnivores such as raccoon dogs ( *Nyctereutes procyonoides* Gray) or badgers ( *Meles leucurus* Hodgson) may differ considerably in attractiveness to dung beetles. Similarly, wild boar dung may be more attractive than other omnivore dung types due to factors unrelated to diet, such as specific volatile compounds, microbial communities, or nutritional compositions (Dormont et al. 2004; Frank et al. 2018; Frank, Brückner, et al. 2017; Tonelli et al. 2021). Future experiments incorporating a broader range of dung types and chemical analyses would help disentangle whether dung beetles respond to general traits associated with feeding guilds or to species‐specific dung characteristics in South Korea.

## 5 Conclusion

This study investigated dung beetle community structures across various land‐use types and dung types using multiple diversity indices to enhance our understanding and inform conservation efforts. The findings revealed that deciduous forests and agricultural fields are priority habitats for dung beetles and that biotic interactions with wild boar are important. These results are consistent with global patterns, as low‐disturbance habitats support high taxonomic and functional diversity of dung beetles (Leandro et al. 2023; Nichols et al. 2007). Additionally, large‐bodied species and tunnelers favored low‐disturbance, forested habitats (Barragán et al. 2011; Larsen et al. 2008), while small‐bodied species and dwellers thrived in more disturbed, anthropogenic environments (Filgueiras et al. 2022; Nichols et al. 2007). The preference for wild boar dung also aligns with findings from other bioregions showing high dung beetle attraction to omnivore dung (Enari et al. 2013; Estrada et al. 1999; Frank et al. 2018; Whipple and Hoback 2012), although mammal‐specific variations may exist. In contrast, rollers in South Korea were highly associated with deciduous forests, differing from other parts of the world, where they often favored open or disturbed habitats (Filgueiras et al. 2022; Kunz and Krell 2011; Lobo 2001). This discrepancy may reflect past extinction events in South Korea.

These findings highlight three key priorities for dung beetle conservation. First, oak‐dominant deciduous forests with low levels of disturbance are critical habitats, supporting high biodiversity and endangered roller and tunneler species. This suggests that the conversion of these forests to coniferous plantations should consider dung beetle diversity during the planning phase. Second, agricultural fields support diverse dung beetle communities, and changes in livestock management may further enhance biodiversity. Third, boar dung is a vital resource for dung beetles, suggesting that the ecological consequences of culling wild boar should be carefully assessed. Together, these insights contribute to a conservation framework for dung beetles grounded in both regional and global ecological patterns, and call for expanded research and habitat protection efforts in South Korea.

## Author Contributions

**Suk Young Hong:** conceptualization (lead), data curation (lead), formal analysis (lead), methodology (equal), visualization (lead), writing – original draft (lead), writing – review and editing (equal). **Minwoo Oh:** data curation (supporting), formal analysis (supporting), visualization (supporting), writing – review and editing (equal). **Yoonjeong Heo:** data curation (supporting), visualization (supporting), writing – review and editing (equal). **Eun Ju Lee:** funding acquisition (lead), supervision (lead), writing – review and editing (equal).

## Conflicts of Interest

The authors declare no conflicts of interest.

## Data Availability

The data were deposited in Dryad under the reference link https://doi.org/10.5061/dryad.mkkwh71bk.

## 1

**TABLE A1:** List of dung beetle species found in this experiment and their occurrence at different land‐use types and dung types.

Species			Built				Coniferous				Deciduous				Field				Grand total
Name	Size	Nesting	Boar	Cattle	Lcat	Total	Boar	Cattle	Lcat	Total	Boar	Cattle	Lcat	Total	Boar	Cattle	Lcat	Total	
*Phelotrupes auratus* Motschulsky, 1858	Large	Tunneler									38	14	4	56					56
*Sisyphus schaefferi* Linnaeus 1758	Medium	Roller					4			4	133	1	17	151					155
*Copris tripartitus* Waterhouse, 1875	Large	Tunneler					20	1		21	86	19	3	108	2			2	131
*Liatongus phanaeoides* Westwood, 1840	Medium	Tunneler									2			2					2
*Caccobius brevis* Waterhouse, 1875	Small	Tunneler	1			1									19			19	20
*Caccobius unicornis* Fabricius, 1798	Small	Tunneler	9			9									3		1	4	13
*Onthophagus japonicus* Harold, 1874	Medium	Tunneler	3	1		4	13		1	14	35			35	21		1	22	75
*Onthophagus lenzii* Harold, 1874	Medium	Tunneler	13	1		14	3			3	3			3	29		3	32	52
*Onthophagus fodiens* Waterhouse, 1875	Medium	Tunneler	65	12	29	106	412	104	196	712	252	105	125	482	70	15	12	97	1397
*Onthophagus atripennis* Waterhouse, 1875	Medium	Tunneler	105	6	47	158	49	5	25	79	82	38	54	174	10		2	12	423
*Onthophagus punctator* Reitter, 1893	Small	Tunneler	3		2	5	7	7		14		1		1	5			5	25
*Onthophagus koryoensis* Kim, 1985	Medium	Tunneler					3	1		4	12	3	3	18	3		1	4	26
*Onthophagus olsoufieffi* Boucomont, 1924	Medium	Tunneler															2	2	2
*Onthophagus rugulosus* Harold, 1885	Large	Tunneler	7			7	31	1	3	35	82	3		85	5			5	132
*Onthophagus bivertex* Heyden, 1887	Medium	Tunneler													6		1	7	7
*Aphodius urostigma* Harold, 1862	Small	Dweller													1			1	1
*Aphodius variabilis* Waterhouse, 1875	Medium	Dweller	3			3	2			2	67		1	68	116		3	119	192
*Aphodius botulus* Balthasar, 1945	Small	Dweller		1		1						1		1					2
*Aphodius rectus* Motschulsky, 1886	Medium	Dweller	5	1		6					1			1	30			30	37
*Aphodius pusillus* Herbst, 1789	Small	Dweller	1			1					2			2	1			1	4
*Aphodius fasciatus* Olivier, 1789	Small	Dweller										3		3		2		2	5
*Aphodius sublimbatus* Motschulsky, 1860	Small	Dweller	1		1	2									72	2	2	76	78
*Saprosites japonicus* Waterhouse, 1875	Small	Dweller														1		1	1
*Psammodiini* sp.	Small	Dweller	3	1	1	5									3		3	6	11
No. of species			13	7	5	14	10	6	4	10	13	10	7	16	17	4	11	20	24
Total abundance			219	23	80	322	544	119	225	888	795	188	207	1190	396	20	31	447	2847

**TABLE A2:** Results of the PERMANOVA by land‐use type and dung type.

PERMANOVA		df	SumofSqs	*R* ^2^	*F*	Pr(>*F*)	Sig.
Land‐use type	Model	3	5.486	0.252	8.196	0.001	***
	Residual	73	16.289	0.748			
	Total	76	21.776	1			
Dung type	Model	2	2.839	0.130	5.546	0.001	***
	Residual	74	18.937	0.870			
	Total	76	21.776	1.000			

*Note:* The analysis was based on Bray–Curtis dissimilarity, and the significance was assessed using 999 permutations.

Sig. codes: ***< 0.001, **< 0.01, *< 0.05, ^#^< 0.1.

**TABLE A3:** Pairwise post hoc analysis of dung beetle richness (Chao1) and abundance by land‐use type and dung type.

Index	Variable	Contrast	Estimate	SE	*z* ratio	*p*	Sig.
Taxonomic richness	Land‐use type	Built–field	−0.0819	0.0914	−0.8952	0.8073	
		Built–coniferous	−0.1139	0.0847	−1.3447	0.5343	
		Built–deciduous	−0.3841	0.0827	−4.6451	< 0.001	***
		Field–coniferous	−0.0321	0.0871	−0.3683	0.9829	
		Field–deciduous	−0.3022	0.0825	−3.6632	0.0014	**
		Coniferous–deciduous	−0.2701	0.0770	−3.5073	0.0026	**
	Dung type	Boar–cattle	0.7609	0.0731	10.4031	< 0.001	***
		Boar–lcat	0.8498	0.0696	12.2067	< 0.001	***
		Cattle–lcat	0.0889	0.0798	1.1140	0.5054	
Taxonomic abundance	Land‐use type	Built–field	0.1556	0.2650	0.5871	0.9360	
		Built–coniferous	−0.7907	0.2627	−3.0101	0.0139	*
		Built–deciduous	−0.9602	0.2607	−3.6832	0.0013	**
		Field–coniferous	−0.9462	0.2755	−3.4345	0.0033	**
		Field–deciduous	−1.1158	0.2707	−4.1213	< 0.001	***
		Coniferous–deciduous	−0.1695	0.2552	−0.6643	0.9105	
	Dung type	Boar–cattle	1.4295	0.1336	10.6992	< 0.001	***
		Boar–lcat	1.0201	0.1154	8.8409	< 0.001	***
		Cattle–lcat	−0.4094	0.1516	−2.6993	0.0190	*

*Note:* The GLMMs were constructed using land‐use type and dung type were used as fixed variables, while month and site ID were used as random variables. The post hoc test was performed on the GLMM. *p*‐value adjustment was conducted using the Tukey test.

Sig. codes: ***< 0.001, **< 0.01, *< 0.05, ^#^< 0.1.

**TABLE A4:** Pairwise post hoc analysis of functional richness (FRic), functional dispersion (FDis), and functional entropy (RaoQ) by land‐use type and dung type.

Index	Variable	Contrast	Estimate	SE	*z* ratio	*p*	Sig.
FRic	Land‐use type	Built–field	0.1363	0.1135	1.2012	0.6260	
		Built–coniferous	0.1080	0.1229	0.8782	0.8162	
		Built–deciduous	−0.3673	0.1229	−2.9883	0.0149	*
		Field–coniferous	−0.0284	0.1227	−0.2312	0.9956	
		Field–deciduous	−0.5036	0.1227	−4.1062	< 0.001	***
		Coniferous–deciduous	−0.4753	0.1310	−3.6278	0.0016	**
	Dung type	Boar–cattle	0.6702	0.1063	6.0751	< 0.001	***
		Boar–lcat	0.6885	0.1062	6.4835	< 0.001	***
		Cattle–lcat	0.0184	0.1062	0.1729	0.9837	
FDis	Land‐use type	Built–field	−0.0196	0.0460	−0.4260	0.9737	
		Built–coniferous	0.1109	0.0547	2.0290	0.1987	
		Built–deciduous	−0.0012	0.0547	−0.0210	1.0000	
		Field–coniferous	0.1305	0.0534	2.4460	0.0886	^#^
		Field–deciduous	0.0184	0.0534	0.3450	0.9856	
		Coniferous–deciduous	−0.1121	0.0572	−1.9580	0.2250	
	Dung type	Boar–cattle	0.0454	0.0337	1.3468	0.3804	
		Boar–lcat	0.0605	0.0337	1.7945	0.1875	
		Cattle–lcat	0.0151	0.0337	0.4478	0.8957	
RaoQ	Land‐use type	Built–field	−0.0103	0.0221	−0.4665	0.9660	
		Built–coniferous	0.0618	0.0272	2.2713	0.1262	
		Built–deciduous	0.0087	0.0272	0.3206	0.9884	
		Field–coniferous	0.0721	0.0267	2.7000	0.0506	^#^
		Field–deciduous	0.0190	0.0267	0.7127	0.8908	
		Coniferous–deciduous	−0.0531	0.0287	−1.8484	0.2706	
	Dung type	Boar–cattle	0.0204	0.0162	1.2580	0.4288	
		Boar–lcat	0.0214	0.0162	1.3222	0.3935	
		Cattle–lcat	0.0010	0.0162	0.0642	0.9977	

*Note:* The GLMMs were constructed using land‐use type and dung type were used as fixed variables, while month and site ID were used as random variables. The post hoc test was performed on the GLMM. *p*‐value adjustment was conducted using the Tukey test.

Sig. codes: ***< 0.001, **< 0.01, *< 0.05, ^#^< 0.1.

**TABLE A5:** Pairwise post hoc analysis of dung beetle richness (Chao1) by land‐use type and dung type, when categorized by size (body length).

Index	Trait	Contrast	Estimate	SE	*z* ratio	*p*	Sig.
Richness land‐use type	Large	Built–field	0.5599	0.6324	0.8853	0.8125	
		Built–coniferous	−1.4950	0.4188	−3.5696	0.0020	**
		Built–deciduous	−2.3269	0.3968	−5.8642	< 0.001	***
		Field–coniferous	−2.0548	0.5328	−3.8564	< 0.001	***
		Field–deciduous	−2.8867	0.5156	−5.5993	< 0.001	***
		Coniferous–deciduous	−0.8319	0.2020	−4.1175	< 0.001	***
	Medium	Built–field	−0.0639	0.1410	−0.4532	0.9690	
		Built–coniferous	−0.0675	0.1341	−0.5034	0.9583	
		Built–deciduous	−0.2429	0.1264	−1.9216	0.2189	
		Field–coniferous	−0.0036	0.1336	−0.0269	1.0000	
		Field–deciduous	−0.1790	0.1251	−1.4304	0.4803	
		Coniferous–deciduous	−0.1754	0.1171	−1.4983	0.4385	
	Small	Built–field	−0.6854	0.2790	−2.4565	0.0669	
		Built–coniferous	0.7896	0.3802	2.0767	0.1607	
		Built–deciduous	1.5631	0.4708	3.3204	0.0050	**
		Field–coniferous	1.4750	0.3469	4.2513	< 0.001	***
		Field–deciduous	2.2485	0.4442	5.0620	< 0.001	***
		Coniferous–deciduous	0.7735	0.5115	1.5121	0.4301	
Richness dung type	Large	Boar–cattle	1.1564	0.2321	4.9816	< 0.001	***
		Boar–lcat	2.0473	0.3349	6.1128	< 0.001	***
		Cattle–lcat	0.8909	0.3819	2.3329	0.0514	^#^
	Medium	Boar–cattle	0.7080	0.1242	5.7001	< 0.001	***
		Boar–lcat	0.5351	0.1105	4.8429	< 0.001	***
		Cattle–lcat	−0.1729	0.1436	−1.2039	0.4508	
	Small	Boar–cattle	0.2308	0.2860	0.8071	0.6986	
		Boar–lcat	1.2358	0.3851	3.2089	0.0038	*
		Cattle–lcat	1.0050	0.4336	2.3177	0.0534	^#^

*Note:* The dung beetles were categorized into small, medium, and large based on their body length. In constructing the GLMMs, land‐use type, dung type, and trait group (size) were used as fixed variables, while month and site ID were used as random variables. The post hoc test was performed on the GLMM, and the *p*‐value adjustment was conducted using the Tukey test.

Sig. codes: ***< 0.001, **< 0.01, *< 0.05, ^#^< 0.1.

**TABLE A6:** Pairwise post hoc analysis of dung beetle abundance by land‐use type and dung type, when categorized by size (body length).

Index	Trait	Contrast	Estimate	SE	*z* ratio	*p*	Sig.
Abundance land‐use type	Large	Built–field	0.2982	0.6280	0.4748	0.9625	
		Built–coniferous	−1.9439	0.5113	−3.8020	< 0.001	***
		Built–deciduous	−3.4448	0.4978	−6.9195	< 0.001	***
		Field–coniferous	−2.2421	0.5193	−4.3179	< 0.001	***
		Field–deciduous	−3.7430	0.5055	−7.4040	< 0.001	***
		Coniferous–deciduous	−1.5009	0.3413	−4.3979	< 0.001	***
	Medium	Built–field	0.0298	0.2868	0.1039	0.9996	
		Built–coniferous	−0.7322	0.2947	−2.4848	0.0623	^#^
		Built–deciduous	−0.7723	0.2923	−2.6420	0.0411	*
		Field–coniferous	−0.7620	0.3034	−2.5118	0.0581	^#^
		Field–deciduous	−0.8021	0.2962	−2.7079	0.0342	*
		Coniferous–deciduous	−0.0402	0.2908	−0.1382	0.9991	
	Small	Built–field	−0.9417	0.3852	−2.4444	0.0690	^#^
		Built–coniferous	0.8709	0.4528	1.9232	0.2182	
		Built–deciduous	1.6528	0.5247	3.1502	0.0089	**
		Field–coniferous	1.8126	0.4260	4.2550	< 0.001	***
		Field–deciduous	2.5945	0.5014	5.1742	< 0.001	***
		Coniferous–deciduous	0.7820	0.5505	1.4204	0.4865	
Abundance dung type	Large	Boar–cattle	1.7872	0.2494	7.1654	< 0.001	***
		Boar–lcat	3.1282	0.3732	8.3814	< 0.001	***
		Cattle–lcat	1.3410	0.4078	3.2888	0.0029	**
	Medium	Boar–cattle	1.2851	0.1513	8.4949	< 0.001	***
		Boar–lcat	0.8024	0.1405	5.7108	< 0.001	***
		Cattle–lcat	−0.4827	0.1645	−2.9351	0.0093	**
	Small	Boar–cattle	0.6057	0.3176	1.9072	0.1366	
		Boar–lcat	1.7212	0.3781	4.5524	< 0.001	***
		Cattle–lcat	1.1155	0.4533	2.4607	0.0369	*

*Note:* The dung beetles were categorized into small, medium, and large based on their body length. In constructing the GLMMs, land‐use type, dung type, and trait group (size) were used as fixed variables, and month was used as a random variable. The post‐hoc test was performed on the GLMM, and the *p*‐value adjustment was conducted using the Tukey test.

Sig. codes: ***< 0.001, **< 0.01, *< 0.05, ^#^< 0.1.

**TABLE A7:** Pairwise post hoc analysis of dung beetle richness (Chao1) by land‐use type and dung type, when categorized by nesting strategy.

Index	Trait	Contrast	Estimate	SE	*z* ratio	*p*	Sig.
Land‐use type	Dweller	Built–field	−0.8878	0.2948	−3.0112	0.0139	*
		Built–coniferous	2.2682	0.7520	3.0162	0.0136	*
		Built–deciduous	0.4644	0.3678	1.2624	0.5870	
		Field–coniferous	3.1560	0.7284	4.3326	< 0.001	***
		Field–deciduous	1.3522	0.3167	4.2699	< 0.001	***
		Coniferous–deciduous	−1.8038	0.7596	−2.3747	0.0820	^#^
	Roller	Built–field	4.8420	64004.5028	0.0001	1.0000	
		Built–coniferous	−18.4159	5680.0124	−0.0032	1.0000	
		Built–deciduous	−20.6067	5680.0124	−0.0036	1.0000	
		Field–coniferous	−23.2579	63751.9424	−0.0004	1.0000	
		Field–deciduous	−25.4487	63751.9424	−0.0004	1.0000	
		Coniferous–deciduous	−2.1908	0.6106	−3.5877	0.0019	**
	Tunneler	Built–field	0.0807	0.1352	0.5970	0.9330	
		Built–coniferous	−0.2521	0.1194	−2.1122	0.1491	
		Built–deciduous	−0.3587	0.1147	−3.1276	0.0095	**
		Field–coniferous	−0.3328	0.1242	−2.6805	0.0369	*
		Field–deciduous	−0.4395	0.1189	−3.6970	0.0012	**
		Coniferous–deciduous	−0.1066	0.1004	−1.0619	0.7128	
Dung type	Dweller	Boar–cattle	0.8896	0.3506	2.5372	0.0301	*
		Boar–lcat	1.4706	0.4063	3.6196	< 0.001	***
		Cattle–lcat	0.5810	0.4990	1.1643	0.4746	
	Roller	Boar–cattle	2.8858	1.0267	2.8109	0.0137	*
		Boar–lcat	0.6784	0.3990	1.7004	0.2050	
		Cattle–lcat	−2.2074	1.0599	−2.0826	0.0934	^#^
	Tunneler	Boar–cattle	0.6469	0.1059	6.1069	< 0.001	***
		Boar–lcat	0.7813	0.1056	7.3957	< 0.001	***
		Cattle–lcat	0.1344	0.1300	1.0340	0.5554	

*Note:* The dung beetles were categorized into dweller, roller, and tunneler based on their nesting strategy. In constructing the GLMMs, land‐use type, dung type, and trait group (size) were used as fixed variables, and month was used as a random variable. The post hoc test was performed on the GLMM, and the *p*‐value adjustment was conducted using the Tukey test.

Sig. codes: ***< 0.001, **< 0.01, *< 0.05, ^#^< 0.1.

**TABLE A8:** Pairwise post hoc analysis of dung beetle abundance by land‐use type and dung type, when categorized by nesting strategy.

Index	Trait	Contrast	Estimate	SE	*z* ratio	*p*	Sig.
Abundance land‐use type	Dweller	Built–field	−2.1226	0.4047	−5.2445	< 0.001	***
		Built–coniferous	2.4227	0.8143	2.9753	0.0155	*
		Built–deciduous	−0.8159	0.4222	−1.9326	0.2143	
		Field–coniferous	4.5453	0.7819	5.8134	< 0.001	***
		Field–deciduous	1.3067	0.3497	3.7362	0.0011	**
		Coniferous–deciduous	−3.2386	0.7878	−4.1111	< 0.001	***
	Roller	Built–field	6.4703	59287.8272	0.0001	1.0000	
		Built–coniferous	−16.9894	2582.6700	−0.0066	1.0000	
		Built–deciduous	−20.2952	2582.6700	−0.0079	1.0000	
		Field–coniferous	−23.4597	59231.5417	−0.0004	1.0000	
		Field–deciduous	−26.7654	59231.5417	−0.0005	1.0000	
		Coniferous–deciduous	−3.3057	0.5994	−5.5148	< 0.001	***
	Tunneler	Built–field	0.4941	0.2773	1.7821	0.2819	
		Built–coniferous	−0.7788	0.2865	−2.7181	0.0332	*
		Built–deciduous	−0.7923	0.2836	−2.7935	0.0268	*
		Field–coniferous	−1.2729	0.2941	−4.3275	< 0.001	***
		Field–deciduous	−1.2864	0.2890	−4.4516	< 0.001	***
		Coniferous–deciduous	−0.0135	0.2802	−0.0482	1.0000	
Dung type	Dweller	Boar–cattle	2.1124	0.3692	5.7220	< 0.001	***
		Boar–lcat	2.5593	0.3772	6.7842	< 0.001	***
		Cattle–lcat	0.4469	0.4926	0.9072	0.6358	
	Roller	Boar–cattle	4.4610	1.0360	4.3058	< 0.001	***
		Boar–lcat	1.7008	0.3581	4.7498	< 0.001	***
		Cattle–lcat	−2.7602	1.0659	−2.5896	0.0260	*
	Tunneler	Boar–cattle	1.1340	0.1608	7.0503	< 0.001	***
		Boar–lcat	0.8209	0.1524	5.3877	< 0.001	***
		Cattle–lcat	−0.3131	0.1783	−1.7560	0.1846	

*Note:* The dung beetles were categorized into dweller, roller, and tunneler based on their nesting strategy. In constructing the GLMMs, land‐use type, dung type, and trait group (size) were used as fixed variables, and month was used as a random variable. The post hoc test was performed on the GLMM, and the *p*‐value adjustment was conducted using the Tukey test.

Sig. codes: ***< 0.001, **< 0.01, *< 0.05, ^#^< 0.1.
